# Editorial: The brain in pain: a multidimensional approach

**DOI:** 10.3389/fpsyg.2024.1401784

**Published:** 2024-04-17

**Authors:** Francesca Benuzzi, Alexa Müllner-Huber, Carlo Adolfo Porro, Fausta Lui

**Affiliations:** ^1^Department of Biomedical, Metabolic and Neural Sciences, University of Modena and Reggio Emilia, Modena, Italy; ^2^Psychology of Ageing Research Unit, Department of Developmental and Educational Psychology, University of Vienna, Vienna, Austria; ^3^Social, Cognitive and Affective Neuroscience Unit (SCAN-Unit), Department of Cognition, Emotion, and Methods in Psychology, University of Vienna, Vienna, Austria

**Keywords:** pain, empathy, Event-Related Potentials (ERPs), Near-InfraRed-Spectroscopy (NIRS), Magnetic Resonance Imaging (MRI), meta-analysis

## Introduction/background

The aim of the Research Topic “*The brain in pain: a multidisciplinary approach*” is to collect the latest quality research on the subject, focusing on the multiple facets of pain in humans, from its neural substrates to its possible expressions and modulation.

We have published papers by a total of 63 Authors, affiliated to research institutions located in five countries in three different continents, employing a variety of techniques, from behavioral, psychological and sensory testing, to Event-Related Potentials (ERPs), Near-InfraRed-Spectroscopy (NIRS) and functional Magnetic Resonance Imaging (fMRI), and including meta-analysis of previous research. Some of the studies dealt with specific chronic pain patients, others with different modulatory factors in healthy volunteers.

## Overview of the articles in this Research Topic

Primary dysmenorrhea (PDM) is a very common cause of pelvic pain in women during their fertile years, with severe consequences for their quality of life (Ferries-Rowe et al., 2020). The study by Lee et al. demonstrates that young Asian PDM females, who, differently from Caucasian PDM patients, do not express pain hypersensitivity, during acute noxious heat stimulation show reduced response and de-coupling of the Default Mode Network (DMN), but only in the painful menstrual phase. Another study by the same research group (Hsu et al.) reveals an influence of the A118G polymorphism of the OPRM1 gene on white matter features, especially of the motor network, but only during the painful menstrual phase, possibly with a maladaptive role. These results offer interesting contributions to the discussion of ethnic, genetic and hormonal influences both on pain perception and on its neural mechanisms.

Two studies focus on fibromyalgia (FM), another chronic pain condition, mostly affecting women (Ruschak et al., 2023). Bao et al. investigated the relationship between fibromyalgia and long-term opioid use, revealing that, quite unexpectedly, temporal summation does not significantly change in FM patients, but negatively correlates with pain ratings, whereas higher opioid dosage correlates with higher heat pain sensitivity. On the other hand, Xin et al. performed a metanalysis of voxel-based morphometry (VBM) studies in FM, including updated data with respect to previous studies (see, e.g., Dehghan et al., 2016). They found changes in gray matter (GM) in FM patients, namely, increased GM in right postcentral gyrus and left angular gyrus, and decreased GM in right cingulate gyrus, right paracingulate gyrus, left cerebellum, and left gyrus rectus, i.e., brain regions involved in different (somatosensory, affective, cognitive) functions. These findings suggest both structural and functional adjustments, in a complex pain syndrome such as fibromyalgia.

Two more studies dealt with different forms of neuropathic pain: Du et al. adopted functional near-infrared spectroscopy (fNIRS) to detect cerebral changes in patients with cervical spondylosis. During acute pain stimulation, they found substantial increases in oxyhemoglobin concentrations in the frontal pole and dorsolateral prefrontal cortex, which significantly decreased in stimulation trials following analgesic procedures; these results add to our knowledge about the role of DLPFC in chronic pain conditions and about its potential as a therapeutic target (Seminowicz and Moayedi, 2017). In the study by Bu et al., spinal cord stimulation not only effectively reduced pain and other anomalies, such as sleep disorders, in patients affected by postherpetic neuralgia, but it also induced both static and dynamic brain resting state activity changes, which in some regions correlate with clinical characteristics.

Pain can be modulated by several factors, including physical exercise and training, although the underlying mechanisms are not yet well understood (Lesnak and Sluka, 2020). Peier et al. compared endurance athletes to non-trained individuals, and identified a pain-resistant population, especially numerous among athletes, who show some peculiarities in their EEG pattern during pain perception (i.e., reduced global power spectra in the beta bands, as opposed to the increase found in non-resistant non-athletes); it is worth pointing out that the characterization of pain responses might lead to a personalized, and therefore more efficient, pain management.

Pain empathy is a powerful means to improve interpersonal communication and prosocial behavior (see, e.g., Smith et al., 2020). The study by Li et al. reveals ERP changes depending on the moral judgment given by the participant on the person experiencing pain, namely, smaller mean wave amplitude of positive 300 (P3) and late positive potential (LPP) for painful pictures of individuals deserving a low moral judgement, and vice versa for people deserving a high moral judgement; notably, the study puts these results in relationship with the issue of violence against healthcare operators, a reason of globally increasing alarm (Banga et al., 2023).

Finally, two studies investigate the intriguing relationship between pain and language (Borelli et al., 2021). Gilioli et al. investigated the electrophysiological correlates of implicit processing of words with pain content using an affective priming paradigm. The study indicates that valence and semantics of a stimulus interact to produce specific emotional responses. This research increases our knowledge of how pain-related words impact cognitive processing and emotional reactions, providing insights into the complex interplay among pain, affective priming, and cognitive mechanisms. Lastly, the study by Borelli et al. presents an event-related fMRI study that aimed to compare brain activity related to perceiving nociceptive pain and processing semantic pain, and specifically, words related to either physical or social pain. The results show that words associated with social pain activate regions linked to affective-motivational aspects of pain perception; conversely, words related to physical pain trigger activity in regions associated with sensory-discriminative aspects of pain perception; the degree of activation in specific regions vary depending on the type of pain being processed. This study sheds light on how words associated with physical and social pain influence the brain networks involved in pain perception.

In conclusion, the present Research Topic, “*The brain in pain: a multidimensional approach*”, brings together cutting-edge research and diverse perspectives to increase our understanding of how the brain perceives, processes, and responds to pain. By doing so, it both advances our knowledge on the neuroscience of pain, and offers new perspectives for innovative approaches to pain management and treatment.

## Author contributions

FB: Writing—review & editing, Writing—original draft, Project administration, Conceptualization. AM-H: Writing—review & editing, Project administration, Conceptualization. CAP: Writing—review & editing, Supervision, Conceptualization. FL: Writing—review & editing, Writing—original draft, Supervision, Project administration, Conceptualization.
